# Work‐related factors and risk of amyotrophic lateral sclerosis: A multivariable Mendelian randomization study

**DOI:** 10.1002/brb3.3317

**Published:** 2023-11-13

**Authors:** Ming Li, Yile Liao, Zhangkun Luo, Hongfei Song, Zhi Yang

**Affiliations:** ^1^ Department of Neurology Changning County Hospital of Traditional Chinese Medicine Yibin China; ^2^ School of Acupuncture and Massage Chengdu University of Traditional Chinese Medicine Chengdu China; ^3^ State Key Laboratory of Southwestern Chinese Medicine Resources School of Basic Medical Sciences Chengdu University of Traditional Chinese Medicine Chengdu China; ^4^ School of Basic Medical Sciences Chengdu University of Traditional Chinese Medicine Chengdu China; ^5^ Department of Respiratory and Critical Care Medicine Changning County Hospital of Traditional Chinese Medicine Yibin China

**Keywords:** amyotrophic lateral sclerosis, job involves heavy manual or physical work, Mendelian randomization, single‐nucleotide polymorphisms

## Abstract

**Background:**

The causal relationship between work‐related factors and amyotrophic lateral sclerosis (ALS) is unclear. We used a Mendelian randomization (MR) analysis to investigate the unconfounded association between work‐related factors and ALS.

**Methods:**

Univariable MR analyses were conducted to evaluate the causal effects of work‐related factors on ALS. Instrumental variables from the UK Biobank on work‐related factors (*n* = 263,615) were used as proxies. The outcome dataset used ALS (*n* case = 20,806, *n* control = 59,804) summary‐level data from a large‐scale genome‐wide association study based on European ancestry. MR analysis used inverse variance weighted (IVW), MR‐Egger, and weighted median (WM) to assess causal effects and other methods of MR for sensitivity analysis. Further multivariable MR analyses were performed to explore potential mediating effects.

**Results:**

In univariable MR, IVW methods support evidence that genetically determined job involves heavy manual or physical work (OR = 2.04, 95% CI: 1.26–3.31; *p* = .004) was associated with an increased risk of ALS, and the WM methods also confirm this result (OR = 2.36, 95% CI: 1.30–4.28; *p* = .005). No evidence of heterogeneity or horizontal pleiotropy was found in the results. In multivariable MR, the association was absent after adjusting for smoking and blood pressure.

**Conclusions:**

Our MR analysis results demonstrate the potential causal relationship between jobs that involve heavy manual or physical work and ALS, which might be mediated by smoking and high systolic blood pressure.

## Graphical abstract

The causal relationship between work‐related factors and amyotrophic lateral sclerosis (ALS) is unclear. Our MR analysis results demonstrate that the causal relationship between job involves heavy manual or physical work on ALS, which might be mediated by smoking and high systolic blood pressure.

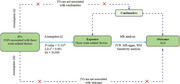

## 1 INTRODUCTION

Amyotrophic lateral sclerosis (ALS) is a common motor neuron disease characterized by progressive damage to upper and lower motor neurons (Brown & Al‐Chalabi, 2017). As the disease progresses, patients with ALS may develop motor dysfunction such as dysarthria, dysphagia, limb weakness, and muscle atrophy; with most of them dying from lung infection or respiratory failure within 2–4 years of disease onset (Chiò et al., 2009). ALS places a significant financial burden on families and society, which will become much severer in the future as the global population ages (Arthur et al., 2016). Given the adverse effects of ALS on individual health and the public health system, it is important to identify the risk factors associated with ALS.

Environmental exposure is a risk factor for many diseases, and as a result, more and more studies are looking at the relationship between work‐related factors and disease. For example, it has been shown that work‐related heavy physical activity increases the risk of dementia (Nabe‐Nielsen et al., 2021). Moe et al. (2013) showed that work involving sedentary work or heavy work increased the risk of metabolic syndrome and cardiovascular mortality compared to people whose work involved a lot of walking/lifting. Although a small number of observational studies have found deleterious effects of work‐related factors on ALS (Beghi et al., 2010; Vanacore et al., 2010), observational studies are unable to draw true causal inferences as the findings are susceptible to reverse causality and confounding factors (Davey Smith & Hemani, 2014).

Randomized controlled trials (RCTs) can infer a causal relationship between exposure and outcome and provide a high level of clinical evidence. However, due to ethical considerations, it is unlikely that an RCT of work‐related factors with ALS could be implemented, so alternative research methods that can mimic RCT are an appropriate option. Mendelian randomization (MR) is an emerging approach to epidemiological research that assesses the causal relationship between exposure and outcome by using single nucleotide polymorphisms (SNPs) as instrumental variables (IVs) (Pierce & Burgess, 2013). MR methods are also known as natural RCTs because the process of assigning alleles from parent to offspring is similar to random grouping in RCT (Davies et al., 2018), and thus, MR methods can overcome some of the limitations in observational studies and minimize confounding factors and reverse causality (Davey Smith & Hemani, 2014). The effect of work‐related factors on some neurodegenerative diseases such as Alzheimer's disease has been demonstrated by MR (Zhao et al., 2023), but there is still a lack of MR studies of work‐related factors on ALS.

Furthermore, several inherent biological hypotheses support the view that work‐related factors increase the risk of developing ALS. First, physical activity associated with heavy physical work may lead to increased oxidative stress and glutamate excitotoxicity (Bastos et al., 2011; Harwood et al., 2009), factors that may play a role in the pathogenesis of ALS (Bastos et al., 2011). Second, there are aggregated protein deposits in the brain tissue of ALS patients (Turner et al., 2013), and shift work may lead to sleep disturbances (Zhao et al., 2021), which in turn reduces the body's clearance of the associated pathological deposits (Winer et al., 2020; Xie et al., 2013). Therefore, based on the above reasons, we propose that work‐related factors may be associated with ALS. Here we designed univariable MR to explore the causal association between work‐related factors and ALS. To identify the potential causal mechanisms linking work‐related factors with ALS, we applied univariable MR to assess genetic associations between work‐related factors and ALS risk factors, such as smoking (Peters et al., 2020), dyslipidemia (low‐density lipoprotein and total cholesterol) (Julian et al., 2022), and blood pressure abnormalities (low diastolic blood pressure and high systolic blood pressure) (Xia et al., 2022). We then used multivariable MR to investigate whether these factors could serve as mediators in the pathway from work‐related factors to ALS. The study may provide sound clinical recommendations for the prevention of ALS.

## 2 METHODS

### 2.1 Dataset

Summary‐level genome‐wide association study (GWAS) data for three work‐related phenotypes were obtained from UK Biobank (Sudlow et al., 2015) and are available by accessing the open Integrative Epidemiology Unit public database (https://gwas.mrcieu.ac.uk/). The three work‐related factors were obtained by completing an online questionnaire. More than 260,000 participants took part in the questionnaire. For “job involves heavy manual or physical work (*n* = 263,615)” and the question was, “Does your work involve heavy manual or physical work?”. Defined as, “physical work includes work that involves the handling of heavy objects and use of heavy tools.” For “job involves shift work (*n* = 263,315),” subjects were asked, “Does your work involve shift work?”. Defined as, “shift work is a work schedule that falls outside of the normal daytime working hours of 9 am–5 pm. This may involve working afternoons, evenings, or nights or rotating through these kinds of shifts.” For “job involves mainly walking or standing (*n* = 263,556),” subjects were asked, “Does your work involve walking or standing for most of the time?”. For each of the previous three questions, subjects were asked to select one of the following six options: “never/rarely,” “sometimes,” “usually,” “always,” “prefer not to answer,” and “do not know.” These options correspond to three different groups; “never/rarely” was used as the control group, “sometimes” was allocated as an intermediate group, and “usually” and “always” served as the different work type groups. “Prefer not to answer” and “do not know” were defined as missing.

We obtained summary‐level data on ALS from a large GWAS study involving 80,610 subjects (*n* case = 20,806, *n* control = 59,804) (Nicolas et al., 2018). Genetic information on cigarettes smoked per day was obtained from the GWAS and Sequencing Consortium of Alcohol and Nicotine Use consortium (Liu et al., 2019). Cigarettes smoked per day were a quasi‐continuous variable described as the number of cigarettes smoked per day by smokers (both current and former smokers). The genetic effect on LDL cholesterol and total cholesterol was obtained from the Global Lipids Genetics Consortium (Willer et al., 2013). LDL cholesterol and total cholesterol were treated as continuous variables. For blood pressure, we used GWAS data on diastolic and systolic blood pressure from the International Consortium of Blood Pressure (Evangelou et al., 2018). Diastolic and systolic blood pressures were regarded as a continuous variable rather than a binary variable considered the presence or absence of hypertension. Population stratification was avoided as both the exposure and the outcome datasets were of European ancestry. The data used in this study did not involve individual level data and therefore did not require additional ethical approval. Details of work‐related factors, ALS and ALS risk factors, are shown in Table S1.

### 2.2 Selection of instrumental variables

**FIGURE 1:**
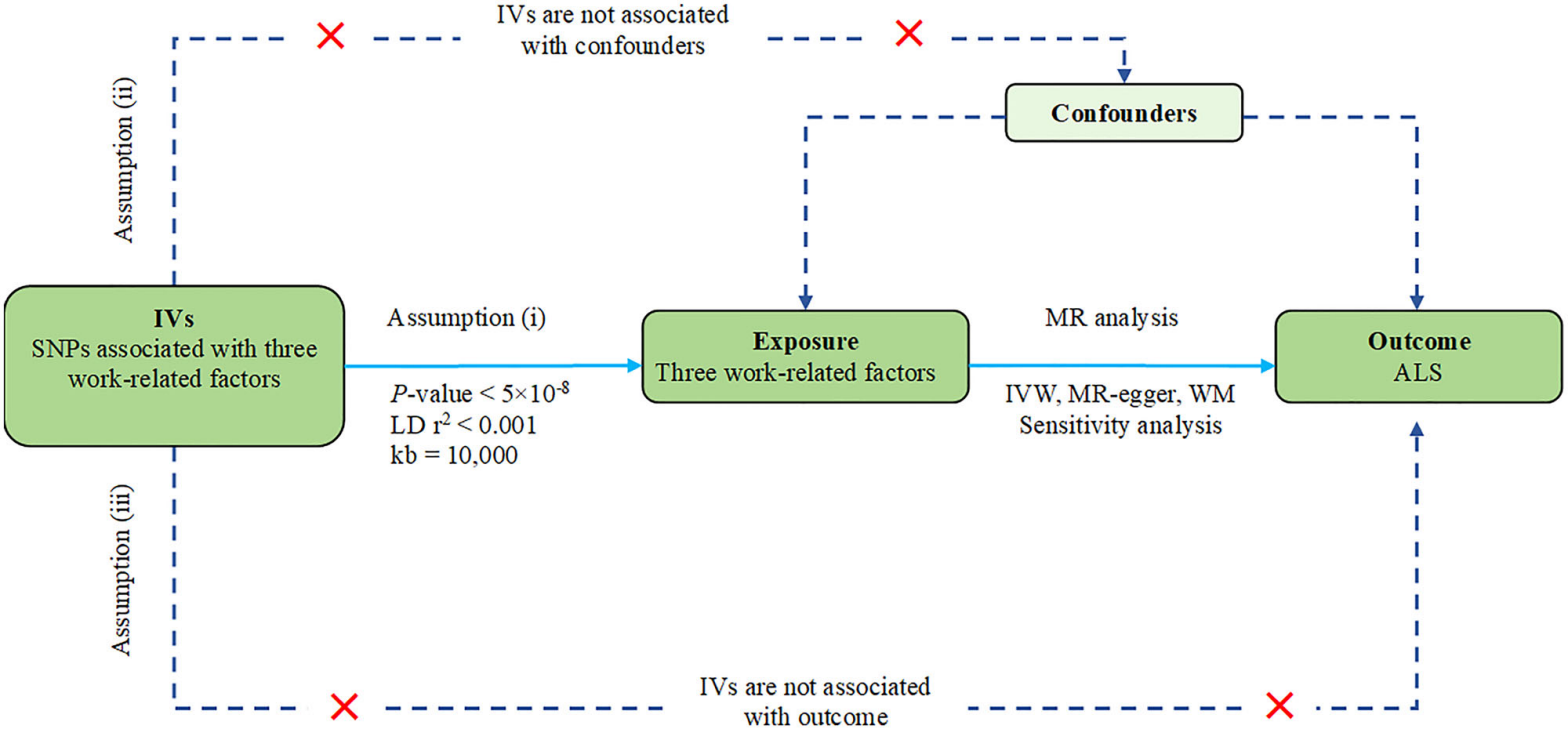
The basic assumptions of Mendelian randomization (MR) study. Assumption (i), instrumental variables (IVs) are strongly related to exposure; assumption (ii), IVs are unrelated to confounders; assumption (iii), IVs are related to outcome only through the exposure of interest. ALS, amyotrophic lateral sclerosis; IVW, Inverse variance weighted; LD, linkage disequilibrium; SNPs, single nucleotide polymorphisms; WM, weighted median.

We hypothesized that work‐related factors are causally related to ALS and that the following three assumptions are met: (i) The IVs are strongly related to work‐related factors; (ii) the IVs are unrelated to any confounding factors that can affect ALS; and (iii) the IVs are related to ALS only through work‐related factors (Figure 1).

We set a series of conditions to pick out the IVs that fit the criteria. First, SNPs that reached a genome‐wide significant level (*p* < 5 × 10^−8^) were selected as IVs and independence between SNPs were maintained by setting the linkage disequilibrium (LD) (*r* ^2^ < .001, kb = 10,000). For the phenotype “job involves shift work,” we only identified two SNPs that strictly met the conditions mentioned above, which limited the feasibility of conducting sensitivity analyses. Therefore, we used a relaxed threshold (*p* < 5 × 10^−6^, *r* ^2^ < .01, kb = 1000), an approach that has been frequently used in previous studies (Choi et al., 2019). Third, for the SNPs that were absent from the ALS dataset, we accessed the SNIPA (https://snipa.helmholtz‐muenchen.de/snipa3/index.php) to identify proxy SNPs at the threshold of Ld *r* ^2^ > .8. If no suitable proxy SNPs were found, we excluded them. Finally, SNPs with palindromic structures were excluded.

Further, we calculated *R* ^2^ and *F* statistics for each SNP. *R* ^2^ indicates the genetic variation explained by each SNP and the *F* statistic reflects the strength of the instrument, whereas SNPs with *F* statistics <10 will be discarded as weak instrumental (Burgess & Thompson, 2011). *R* ^2^ is calculated as follows: $R^{2}=\frac{2\;\times \;EAF\;\times \left(1\;-\;EAF\right)\;\times \;\beta^{2}\;}{SE^{2}\;\times N}\;$ (Wu et al., 2020), where EAF is the effect allele frequency for each SNP, *β* is the allele effect value, SE is the standard error, and *N* is the sample size. *F* statistics is calculated as $F\;=\;\frac{N-K\;-1}{K}\;\times \;\frac{R^{2}}{1\;-\;R^{2}\;}\;$(Burgess & Thompson, 2011), where *N* is the sample size, and *K* is the number of SNPs. Finally, 18, 10, and 13 SNPs were included for MR analysis in job involves heavy manual or physical work, job involves shift work and job involves mainly walking or standing, respectively. Information about SNPs are listed in Tables S2–S4.

### 2.3 Mendelian randomization analysis

For univariable MR analysis, we used inverse variance weighted (IVW) as the primary MR analysis method. IVW is the most commonly used MR method, which combines the effects of individual SNPs on outcomes and calculates overall Wald ratios to assess the association between exposure and outcome (Pierce & Burgess, 2013). The premise of applying IVW is to ensure that all IVs are valid, making IVW the most credible method in MR, but with the disadvantage of not being able to exclude the interference caused by pleiotropy. In addition, MR‐Egger regression and weighted median (WM) were used as complementary methods to IVW to assess causal effects. MR‐Egger allows for the presence of pleiotropy for all IVs, provided that the pleiotropy of IVs is independent of the effect of IVs on exposure (Bowden et al., 2015). When at most half of the invalid IVs are present, WM can still provide robust estimates of causal effects (Bowden et al., 2016). Thus, MR‐Egger and WM can be applied under a wider range of conditions and make up for the shortcomings of the IVW method but have the disadvantage of insufficient statistical power. In addition, we used an online website https://shiny.cnsgenomics.com/mRnd/ to calculate the statistical power of the present study. Using the Bonferroni‐corrected threshold of *p*, *p* < .017 (*α* = .05/3) was considered significant in our study, and *p* between .017 and .05 was considered suggestive of significance.

We then conducted sensitivity analyses to determine whether the MR assumptions were violated. MR‐Egger regression was used to assess the pleiotropy bias caused by IVs. The intercept of MR‐Egger regression can reflect the magnitude of pleiotropy (Bowden et al., 2015). Cochran's *Q* test was used to identify heterogeneity in IVs, and IVs were considered to be without heterogeneity when Cochran Q‐derived *p* value >.05. We used the MR pleiotropy residual sum and outlier (MR‐PRESSO) test to identify outliers with horizontal pleiotropy, and if outliers were present, a corrected MR analysis was performed after removing the outliers to exclude bias from outliers (Verbanck et al., 2018). Finally, we used leave‐one‐out analysis to exclude the strong influence of individual SNPs on the results.

For multivariable MR analysis, we corrected for the effect of ALS risk factors. We first performed univariable MR to explore the causal impact of work‐related factors on ALS risk factors. Subsequently, multivariable MR was performed to assess whether the identified risk factors mediated the observed associations. IVW, WM, and Egger regression were used in the multivariable MR analysis. Heterogeneity of the IVW method was evaluated using the Q‐statistic, whereas polytropy was assessed through the Egger regression intercept.

All analyses were performed in *R* (version 4.2.2) by the packages TwoSampleMR, MRPRESSO, and MendelianRandomization.

## 3 RESULT

### 3.1 Univariable MR analysis

**FIGURE 2:**
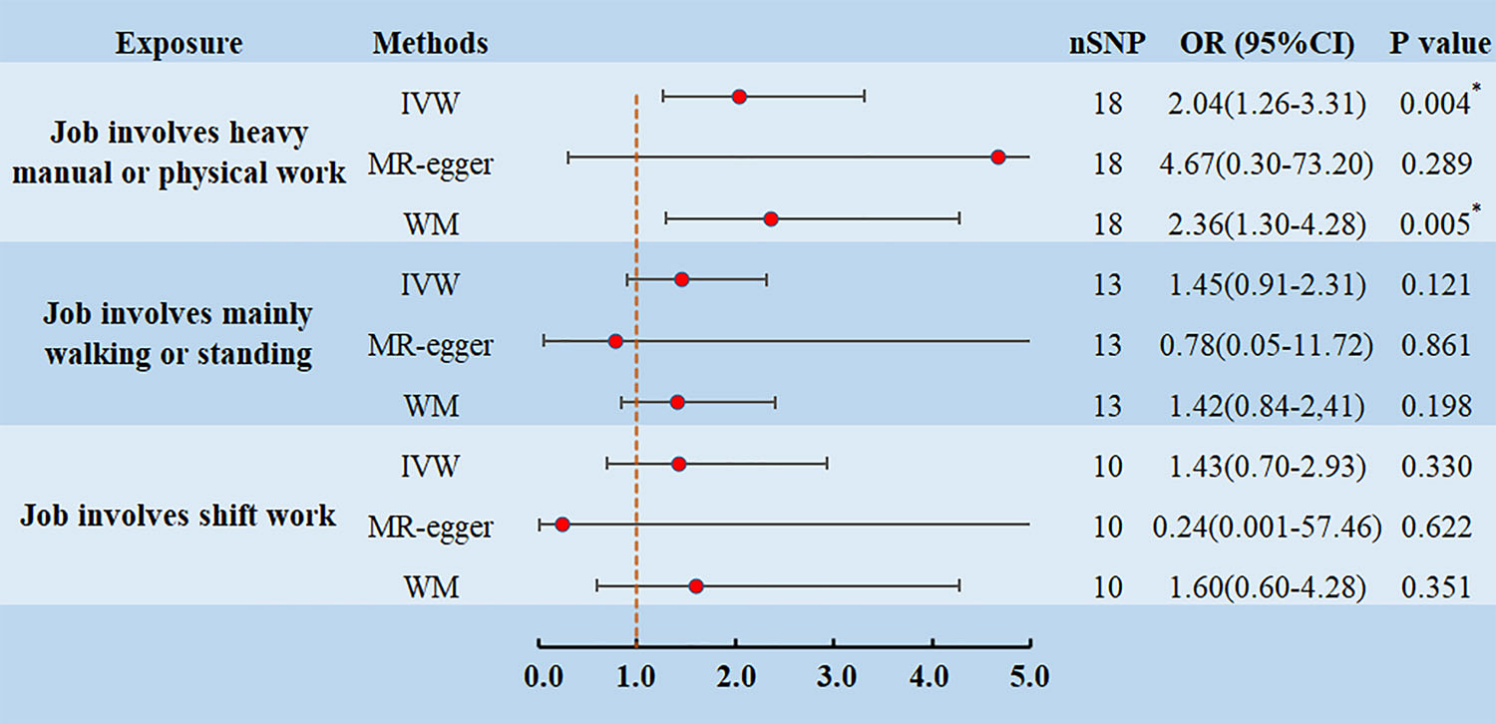
Mendelian randomization (MR) estimates for work‐related factors on amyotrophic lateral sclerosis (ALS) and the forest plot. *Significant is defined as *P*_‐IVW_ < 0.017. CI, confidence intervals; IVW, inverse variance weighted; OR, odds ration; SNP, single‐nucleotide polymorphisms; WM, weighted median.

Among the phenotypes of work‐related factors participating in the study, job involves heavy manual or physical work increased the risk of ALS according to the IVW results (OR = 2.04, 95% CI: 1.26–3.31; *p* = .004 < .017), similar results were obtained with the WM method (OR = 2.36, 95% CI: 1.30–4.28; *p* = .005 < .017), with MR‐egger was not significant but provided consistent direction (Figure 2). For the IVs used in the MR analysis, the calculated *R^2^* was .0014, showing that these SNPs explained about 0.14% of the genetic variation. The overall *F* statistic was 18.09, and all *F* statistics were greater than 10 (ranging from 10.60 to 42.39), indicating that the causal effects of the results were not influenced by weak instruments (Table S2). Given a sample size of 80,610, the proportion of cases was 25.81%, and the significance level *α* was .05. The statistical power based on the calculated OR and *R* ^2^ was 98%. In addition, no causal relationship was found between job involves mainly walking or standing, and job involves shift work, on ALS.

To test the reliability of the results, further sensitivity analyses were carried out. This included the MR‐Egger intercept test, Cochran's *Q* test, leave‐one‐out analysis, and the MR‐PRESSO global test. For the three work‐related factors, all *p* values of the MR‐Egger intercept tests were >.05, excluding interference from horizontal pleiotropy. All *p* values for Cochran's *Q* test analysis were >.05, indicating that there was no heterogeneity in the results (Table 1 and Figure S1). The leave‐one‐out analysis showed no single SNP affecting the results (Figure S2). No outliers were found by the MR‐PRESSO method.

**TABLE 1:** Sensitivity analysis of the causal association between work‐related factors on amyotrophic lateral sclerosis (ALS).

	Cochran's *Q* test		MR‐Egger			MR‐PRESSO global test, *p* value
Exposure	*Q* value	*p* Value	Intercept	Se	*p* Value	
Job involves heavy manual or physical work	23.498	.134	−0.014	0.023	.558	.156
Job involves mainly walking or standing	19.210	.084	0.014	0.030	.659	.130
Job involves shift work	5.950	.745	0.022	0.034	.537	.725

Abbreviation: MR, Mendelian randomization.

### 3.2 Multivariable MR analysis

For multivariable MR, adjusting for the risk factors of ALS, we first performed univariable MR and identified that job involves heavy manual or physical work was causally associated with increased systolic blood pressure (IVW: *β* = 2.16, 95% CI: .71–3.60; *p* = 3.51 × 10^−3^), and increased heaviness of smoking (IVW: *β* = .31, 95% CI: .10–.51; *p* = 3.62 × 10^−3^) (Table 2). With adjustment for smoking and systolic blood pressure, job involves heavy manual or physical work had no causal effect on the increased risk of ALS (Table 3). No positive results were found in the sensitivity analysis.

**TABLE 2:** The causal effects from work‐related factors to the risk factors of the amyotrophic lateral sclerosis (ALS).

		IVW		Cochran's *Q* test		MR‐Egger	
Exposure	Outcome	*β* (95% CI)	*p* Value	*Q* value	*p* Value	Intercept	*p* Value
Job involves heavy manual or physical work	Cigarettes smoked per day	.31(.10, .51)	3.62 × 10^−3*^	20.817	.143	0.003	.786
	LDL cholesterol	.02(−.16, .19)	.847	8.824	.786	−0.001	.493
	Total cholesterol	.05 (−.16, .25)	.662	23.183	.057	0.016	.073
	Systolic blood pressure	2.16 (.71, 3.60)	3.51 × 10^−3*^	21.529	.063	0.117	.367
	Diastolic blood pressure	−.28 (−1.13, .58)	.527	27.052	.019	0.104	.155

Abbreviations: IVW, inverse variance weighted; LDL, low‐density lipoprotein; MR, Mendelian randomization.

**TABLE 3:** Multivariable Mendelian randomization (MR) study results.

		Causal effect		Heterogeneity	Pleiotropy	
Adjustments	Methods	OR (95% CI)	*p* Value	*p* Value	Intercept	*p* Value
Cigarettes smoked per day	IVW	.90 (.80, 1.01)	.065	.540	−0.005	.587
	Median	.94 (.81, 1.10)	.444			
	Egger	.96 (.83, 1.10)	.549			
Systolic blood pressure	IVW	1.00 (1.00, 1.01)	.323	.080	−0.001	.077
	Median	1.00 (.99, 1.01)	.582			
	Egger	1.01 (.99, 1.02)	.355			

Abbreviations: CI, confidence interval; IVW, inverse variance weighted; OR, odds ratio.

## 4 DISCUSSION

Despite ALS patients having a lower quality of life and survival time, unfortunately, we still know very little about how to prevent it (Brown & Al‐Chalabi, 2017). Therefore, we conducted an MR analysis to investigate the causal relationship between three work‐related factors and ALS. Our study found that job involves heavy manual or physical work was risk factor for ALS, with no evidence that the study outcomes were influenced by heterogeneity and pleiotropy. As the statistical power of this study was 98%, it was unlikely to have a false positive result. In addition, for job involves mainly walking or standing and job involves shift work, there was no evidence of a causal relationship between these two work‐related factors and ALS. Further multivariable MR analysis hinted that the observed causal effect might be driven by smoking and high systolic blood pressure. Overall, our findings contribute to the development of scientific early screening strategies and health education for ALS.

As awareness of the disease increases, more researchers are aware of the positive and negative impacts of modifiable work‐related factors on disease. Work‐related factors have been recognized to influence the development of neurodegenerative diseases including dementia, Alzheimer's disease, and ALS. It has been shown that manual work is positively associated with the risk of developing Alzheimer's disease (Zhao et al., 2023). The risk of all‐cause dementia is increased in the shift‐working population (Liao et al., 2022). However, whether physical work plays a harmful role in ALS has been the subject of much debate (Beghi et al., 2010; Vanacore et al., 2010; Veldink et al., 2005). Our study supports the view of Beghi and Vanacore et al. that the risk of ALS is higher in people with heavy physical work. To our knowledge, our study is the first attempt to use MR methods to elucidate the relationship between work‐related factors and ALS.

In addition, due to the lack of research on work‐related factors in the pathogenesis of ALS, we can only speculate about the mechanisms through which heavy physical work may contribute to the development of ALS. Given the strenuous physical activity characteristic of heavy physical work, heavy physical work may fit within the primary hypotheses of ALS pathogenesis: increased oxidative stress and glutamate excitotoxicity (Bastos et al., 2011). First, physical activity can disrupt the balance between the production of free radicals and their removal, leading to increased oxidative stress (Sjödin et al., 1990). Second, physical activity can enhance tissue metabolism, potentially increasing the neurotoxin's potency (Longstreth et al., 1991). Moreover, individuals engaged in heavy physical work are at a higher risk of experiencing trauma, which is considered one of the risk factors for ALS (Pupillo et al., 2012).

Although there are still many unknowns about the pathogenesis of ALS, some influencing factors such as smoking, dyslipidemia, and blood pressure have been confirmed to be involved in the development of ALS. Smoking increases levels of lipid hydroperoxides in serum and cerebrospinal fluid (Anand et al., 2014) and inhibits paraoxonase expression, allowing for increased damage to the body from oxidative stress, which in turn contributes to the development of ALS. There are few studies on the pathogenesis of LDL and ALS, and metabolic abnormalities caused by apolipoprotein B (apoB) are a possible explanation. High levels of apoB are associated with increased oxidative stress in the body, which is common in ALS disease progression (Barber et al., 2006). ApoB can also cause neuronal loss by downregulating vascular endothelial growth factor receptor 1, whereas VEGF has been reported to be associated with neuronal degeneration in ALS patients (Lambrechts et al., 2003). Xia et al. (2022) found a causal relationship among low diastolic blood pressure, high systolic blood pressure, and ALS. The combination of low diastolic and high systolic blood pressure can cause hypoperfusion in the brain, which can disrupt the blood–brain barrier and cause neuroinflammation, accelerating the neurodegenerative process, ultimately promoting the development of ALS (Daulatzai, 2017). Cardiovascular disease caused by blood pressure abnormalities may also be associated with ALS.

To identify mediators between job involves heavy manual or physical work and ALS, we performed multivariable MR analyses. Our results suggest that job involves heavy manual or physical work increases the number of cigarettes smoked per day. In fact, some observational studies have also found an association between work and smoking. For example, work with high physical exertion is associated with heavy smoking (Dobson et al., 2018). Importantly, our multivariable MR analyses provided additional evidence that smoking acts as a mediator for the causal relationship between job involves heavy manual or physical work and ALS. Smokers, especially those whose job involve heavy manual or physical work should be given more attention. Moreover, our results also suggest that job involves heavy manual or physical work increases systolic blood pressure, which is consistent with several previous studies (Clays et al., 2012). After adjusting for systolic blood pressure through multivariable MR analysis, we found that job involves heavy manual or physical work was not causally linked to an increased risk of ALS. This suggests that high systolic blood pressure may also play a role in the relationship between job involves heavy manual or physical work and ALS. Therefore, systolic blood pressure should not be ignored in the population of jobs involving heavy manual or physical work, as it is crucial for preventing ALS.

However, there were still some limitations to our study. First, in the dataset we used, almost all of the subjects were of European ancestry. Therefore, the findings may be less applicable to other racial groups. Second, because the MR hypothesis is based on a linear relationship between exposure and outcome, we were unable to confirm a nonlinear association between work‐related factors and ALS. Third, the incidence of ALS varies across age and gender, but for the protection of the subjects, it was not possible to obtain individual‐level GWAS data to test the effect of gender, and age in work‐related factors on ALS. Finally, given the diversity of ALS patients, work‐related factors may be causally related to specific ALS subtypes, and in‐depth studies of ALS subtype subgroups should be considered in the future.

## 5 CONCLUSION

In conclusion, using large‐scale GWAS summary data, our MR study identifies that job involves heavy manual or physical work increases the risk of ALS, which might be mediated by smoking and systolic blood pressure. More high‐quality observational studies are needed in the future to decipher the underlying mechanisms between work‐related factors and ALS.

## AUTHOR CONTRIBUTIONS

**Ming Li**: Data curation; methodology; writing—original draft; writing—review and editing.**Yile Liao**: Methodology; writing—original draft; writing—review and editing. **Zhangkun Luo**: Writing—original draft; writing—review and editing **Hongfei Song**: Data curation; methodology; writing—review and editing. **Zhi Yang**: Data curation; software; visualization

## CONFLICT OF INTEREST STATEMENT

The authors declare no conflicts of interest.

## FUNDING INFORMATION

This research received no external funding.

### PEER REVIEW

The peer review history for this article is available at https://publons.com/publon/10.1002/brb3.3317.

## Supporting information

Supp Information

## Data Availability

The summary‐level genome‐wide association study (GWAS) data for this study can be found in the Integrative Epidemiology Unit (IEU) public database (https://gwas.mrcieu.ac.uk/).
