# Supplementary material for: Work‐related factors and risk of amyotrophic lateral sclerosis: A multivariable Mendelian randomization study
Source: Brain Behav. 2023 Nov 13;13(12):e3317. doi: 10.1002/brb3.3317 (PMC10726785; doi:10.1002/brb3.3317)
Supplement: Supplementary file 1 — Supp Information [file BRB3-13-e3317-s001.docx]

Supplementary Material

Work-related Factors and Risk of Amyotrophic Lateral Sclerosis: A Two-sample Mendelian Randomization Analysis Based

Table S1. Details of the GWAS included in the Mendelian randomization

Table S2. Instrument variables of job involves heavy manual or physical work

Table S3. Instrument variables of job involves shift work

Table S4. Instrument variables of job involves mainly walking or standing

Figure S1. Causal relationships between work-related factors on ALS in scatterplots

Figure S2. Leave-one-out plots in Mendelian randomization analyses of three work-related factors on ALS

| **Table S1. Details of the GWAS included in the Mendelian randomization.** | | | | | | |
| --- | --- | --- | --- | --- | --- | --- |
| **Traits** |  | **Consortium/Dataset** | **Sample size** | **Sex** | **Ancestry** | **GWAS ID/PMID** |
| Job involves heavy manual or physical work |  | UK Biobank | 263,615 | Mix | European | ukb-b-2002 |
| Job involves mainly walking or standing |  | UK Biobank | 263,556 | Mix | European | ukb-b-4461 |
| Job involves shift work |  | UK Biobank | 263,315 | Mix | European | ukb-b-1712 |
| Amyotrophic lateral sclerosis |  | A meta-analysis of GWAS | 20,806 cases and  59,804 controls | Mix | European | 29566793 |
| Cigarettes smoked per day |  | GSCAN | 249,752 | Mix | European | 30643251 |
| LDL cholesterol |  | GLGC | 173,082 | Mix | European | 24097068 |
| Total cholesterol |  | GLGC | 187,365 | Mix | European | 24097068 |
| Systolic blood pressure |  | ICBP | 757,601 | Mix | European | 30224653 |
| diastolic blood pressure |  | ICBP | 757,601 | Mix | European | 30224653 |

LDL, low-density lipoprotein. GSCN, GWAS and Sequencing Consortium of Alcohol and Nicotine Use. GLGC, Global Lipids Genetics Consortium. SSCAG, Social Science Genetic

Association Consortium. ICBP, International Consortium of Blood Pressure

| **Table S2. Instrument variables of job involves heavy manual or physical work** | | | | | | | | |  |
| --- | --- | --- | --- | --- | --- | --- | --- | --- | --- |
| SNP | Nearest Gene | effect_allele | other_allele | eaf | beta | se | p | R^2^ | F |
| rs2091329 | CYB561D1 | G | A | 0.288157 | -0.01589 | 0.00265 | 2.00E-09 | 5.59E-05 | 14.75 |
| rs12089815 | BARHL2 | A | G | 0.548546 | -0.015147 | 0.002407 | 3.10E-10 | 7.44E-05 | 19.61 |
| rs2819348 | LMOD1 | C | T | 0.352247 | 0.014558 | 0.002497 | 5.60E-09 | 5.88E-05 | 15.51 |
| rs6544763 | CAMKMT | C | T | 0.660134 | 0.013969 | 0.002526 | 3.20E-08 | 5.21E-05 | 13.73 |
| rs11678979 | LINC01104 | C | T | 0.271768 | -0.014993 | 0.002706 | 3.00E-08 | 4.61E-05 | 12.15 |
| rs35999162 | BSN | G | C | 0.31042 | -0.02486 | 0.002587 | 7.40E-22 | 1.50E-04 | 39.53 |
| rs2318540 | AC112518.3 | G | T | 0.572308 | -0.014283 | 0.002422 | 3.70E-09 | 6.46E-05 | 17.02 |
| rs11726786 | TET2 | G | T | 0.366352 | 0.016595 | 0.002483 | 2.40E-11 | 7.86E-05 | 20.73 |
| rs1081158 | CTC-467M3.1 | T | C | 0.581826 | 0.016989 | 0.00243 | 2.70E-12 | 9.02E-05 | 23.79 |
| rs4580876 | RP11-436D23.1 | A | G | 0.476133 | -0.022072 | 0.002395 | 3.00E-20 | 1.61E-04 | 42.39 |
| rs11756123^a^ | ESR1 | T | A | 0.634651 | -0.013749 | 0.002487 | 3.20E-08 | 5.38E-05 | 14.18 |
| rs4731992 | EXOC4 | G | A | 0.781718 | -0.017041 | 0.002895 | 4.00E-09 | 4.49E-05 | 11.82 |
| rs13250996^a^ | SGCZ | C | G | 0.50399 | -0.013312 | 0.002393 | 2.70E-08 | 5.87E-05 | 15.47 |
| rs10820625 | HABP4 | C | T | 0.220898 | -0.016007 | 0.002885 | 2.90E-08 | 4.02E-05 | 10.60 |
| rs7467480 | RP11-370B11.4 | A | T | 0.415774 | -0.015509 | 0.002429 | 1.70E-10 | 7.51E-05 | 19.80 |
| rs7108077 | MAML2 | G | A | 0.379639 | -0.01344 | 0.002463 | 4.90E-08 | 5.32E-05 | 14.02 |
| rs1370059 | RNA5SP30 | G | A | 0.232046 | 0.018082 | 0.002835 | 1.80E-10 | 5.50E-05 | 14.50 |
| rs8054111 | PKD1L3 | G | A | 0.729849 | -0.015349 | 0.002693 | 1.20E-08 | 4.86E-05 | 12.81 |
| rs3785354 | NUPR1 | T | C | 0.367566 | 0.013582 | 0.002467 | 3.70E-08 | 5.35E-05 | 14.09 |
| rs11663824 | DCC | A | C | 0.396798 | 0.013638 | 0.002448 | 2.50E-08 | 5.64E-05 | 14.86 |
| Total |  |  |  |  |  |  |  | 1.37E-03 | 18.09 |
| ^a^SNPs with palindromic structures have been removed from the dataset for amyotrophic lateral sclerosis. | | | | | | | | | |

| **Table S3. Instrument variables of job involves shift work** | | | | | | | | |
| --- | --- | --- | --- | --- | --- | --- | --- | --- |
| SNP | effect_allele | other_allele | eaf | beta | se | p | R^2^ | F |
| rs13019832 | A | G | 0.415708 | -0.013238 | 0.002331 | 1.40E-08 | 5.95E-05 | 15.67 |
| rs13009008 | G | A | 0.671752 | -0.011965 | 0.002436 | 9.00E-07 | 4.04E-05 | 10.64 |
| rs10932655 | C | A | 0.450518 | -0.010706 | 0.002304 | 3.40E-06 | 4.06E-05 | 10.69 |
| rs10865397 | G | A | 0.523443 | -0.011909 | 0.002334 | 3.30E-07 | 4.93E-05 | 12.99 |
| rs59815219 | T | C | 0.494265 | 0.010588 | 0.002291 | 3.80E-06 | 4.05E-05 | 10.68 |
| rs152603 | G | A | 0.366346 | -0.011318 | 0.002376 | 1.90E-06 | 4.00E-05 | 10.53 |
| rs1487441 | A | G | 0.484375 | -0.016149 | 0.002301 | 2.20E-12 | 9.35E-05 | 24.62 |
| rs1860826 | A | G | 0.358721 | -0.011902 | 0.002401 | 7.10E-07 | 4.30E-05 | 11.31 |
| rs4352868 | T | C | 0.43746 | 0.010644 | 0.002315 | 4.30E-06 | 3.95E-05 | 10.41 |
| rs28613960 | A | G | 0.454628 | -0.011486 | 0.002307 | 6.40E-07 | 4.67E-05 | 12.30 |
| Total |  |  |  |  |  |  | 4.93E-04 | 12.99 |

| **Table S4. Instrument variables of job involves mainly walking or standing** | | | | | | | | |
| --- | --- | --- | --- | --- | --- | --- | --- | --- |
| SNP | effect_allele | other_allele | eaf | beta | se | p | R^2^ | F |
| rs11264886 | A | G | 0.283409 | 0.019283 | 0.003379 | 1.10E-08 | 5.02E-05 | 13.23 |
| rs10922907^a^ | T | A | 0.548808 | -0.01908 | 0.003061 | 4.50E-10 | 7.31E-05 | 19.26 |
| rs13019832 | A | G | 0.415716 | -0.01868 | 0.003091 | 1.50E-09 | 6.73E-05 | 17.74 |
| rs9836291 | A | G | 0.294288 | -0.02724 | 0.003337 | 3.30E-16 | 1.05E-04 | 27.67 |
| rs7661349 | C | T | 0.634347 | -0.02016 | 0.003153 | 1.60E-10 | 7.20E-05 | 18.97 |
| rs6882046 | G | A | 0.267534 | -0.02506 | 0.003467 | 4.90E-13 | 7.77E-05 | 20.48 |
| rs9341742 | T | C | 0.399074 | 0.017441 | 0.003107 | 2.00E-08 | 5.73E-05 | 15.11 |
| rs1487445 | T | C | 0.482083 | -0.03071 | 0.003046 | 6.80E-24 | 1.93E-04 | 50.75 |
| rs4731992 | G | A | 0.781756 | -0.02231 | 0.00368 | 1.30E-09 | 4.76E-05 | 12.54 |
| rs603625 | A | G | 0.419262 | -0.01841 | 0.003076 | 2.20E-09 | 6.62E-05 | 17.45 |
| rs6603030 | G | A | 0.794014 | -0.021 | 0.003762 | 2.40E-08 | 3.87E-05 | 10.19 |
| rs3785354 | T | C | 0.367568 | 0.018127 | 0.003136 | 7.50E-09 | 5.89E-05 | 15.53 |
| rs8054111 | G | A | 0.729851 | -0.02126 | 0.003423 | 5.30E-10 | 5.77E-05 | 15.20 |
| rs613872 | T | G | 0.825015 | 0.023602 | 0.004007 | 3.90E-09 | 3.80E-05 | 10.02 |
| Total |  |  |  |  |  |  | 1.00E-03 | 18.88 |

^a^SNPs with palindromic structures have been removed from the dataset for amyotrophic lateral sclerosis.


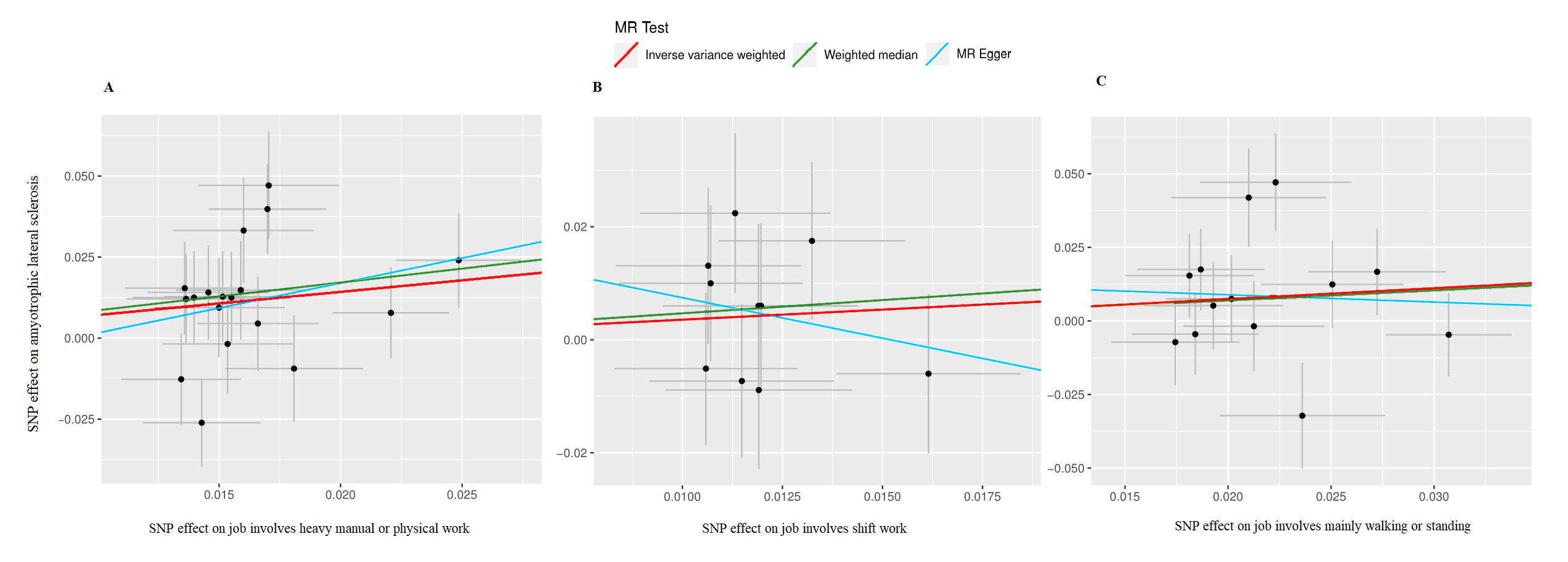


**Figure S1** Causal relationships between work-related factors on ALS in scatterplots. A: genetically predicted job involves heavy manual or physical work on ALS; B: genetically predicted job involves shift work on ALS; C: genetically predicted job involves mainly walking or standing on ALS. SNP, single-nucleotide polymorphisms; ALS, amyotrophic lateral sclerosis


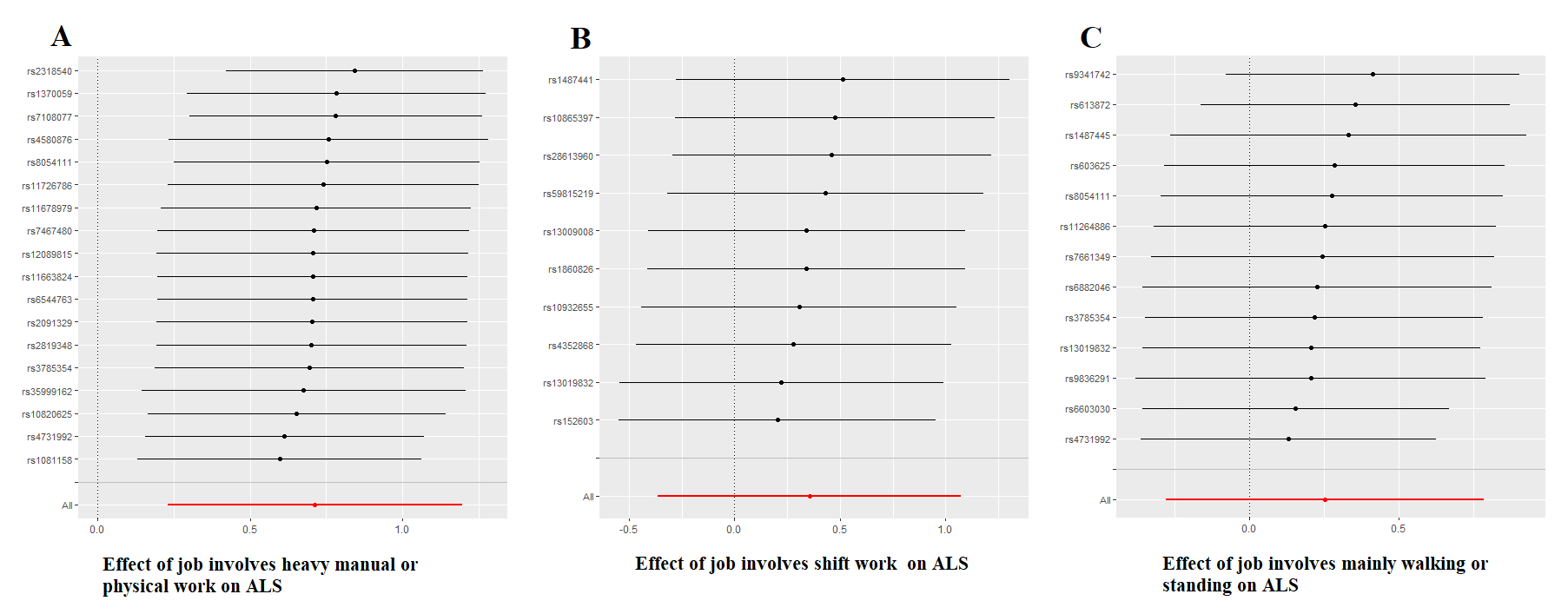


**Figure S2.** Leave-one-out plots in Mendelian randomization analyses of three work-related factors on ALS. A: leave-one-out analysis for job involves heavy manual or physical work on ALS. B: leave-one-out analysis for job involves shift work on ALS. C: leave-one-out analysis for job involves mainly walking or standing on ALS. ALS, amyotrophic lateral sclerosis
